# Correction to "A quick pipeline for the isolation of 3D cell culture‐derived extracellular vesicles"

**DOI:** 10.1002/jev2.12374

**Published:** 2023-10-18

Kyykallio, H., Faria, A. V. S., Hartman, R., Capra, J., Rilla, K., & Siljander, P. R.‐M. (2022). A quick pipeline for the isolation of 3D cell culture‐derived extracellular vesicles. Journal of Extracellular Vesicles, 11, e12273. https://doi.org/10.1002/jev2.12273

In the originally published article, author Rosabella Hartman's last name and email address was spelled incorrectly. This has been corrected in the online version of the article.

We apologize for this error.

